# Circulating Antibodies Against DSG1 and DSG3 in Patients with Oral Lichen Planus: A Scoping Review

**DOI:** 10.3390/antib14020051

**Published:** 2025-06-18

**Authors:** Domenico De Falco, Francesca Iaquinta, Doriana Pedone, Alberta Lucchese, Dario Di Stasio, Massimo Petruzzi

**Affiliations:** 1 Interdisciplinary Department of Medicine, University of Bari, 70121 Bari, Italy; francescaiaquinta00@gmail.com (F.I.); dorianaped98@gmail.com (D.P.); massimo.petruzzi@uniba.it (M.P.); 2 Multidisciplinary Department of Medical and Dental Specialties, University of Campania—“Luigi Vanvitelli”, Via L. De Crecchio, 6, 80138 Naples, Italy; alberta.lucchese@unicampania.it (A.L.); dario.distasio@unicampania.it (D.D.S.)

**Keywords:** oral lichen planus, desmoglein 1, desmoglein 3, OLP, erosive OLP, bullous OLP

## Abstract

Oral Lichen Planus (OLP) is a chronic autoimmune disease with potential overlap with Pemphigus Vulgaris (PV), particularly in erosive forms. Desmoglein 1 and 3 are transmembrane glycoproteins of desmosomes, typically involved in PV. This scoping review aims to evaluate the presence and potential pathogenetic role of anti-desmoglein 1 (Dsg1) and anti-desmoglein 3 (Dsg3) antibodies in OLP. A literature search was conducted on MEDLINE/PubMed, Ovid, and Scopus up to April 2025. Human studies reporting OLP patients with anti-Dsg1 and/or anti-Dsg3 antibodies were included. Data from 11 studies were analyzed by diagnosis, age/sex, oral site involvement, immunofluorescence, and ELISA testing. Erosive OLP was most frequently associated with anti-Dsg1/Dsg3 positivity, mainly in women aged 40–60. Immunofluorescence was positive in some cases, while the ELISA test almost consistently detected anti-Dsg1 and Dsg3 antibodies. However, in many instances, antibody titers did not reach the threshold value, despite the presence being detectable. This finding suggests that anti-Dsg1/Dsg3 antibodies may represent epiphenomena of chronic inflammation in erosive OLP, indicating an immune-serological overlap with PV but lacking direct pathogenicity. Furthermore, the role of Dsg3 in oral squamous cell carcinoma, by promoting enzymes that degrade the extracellular matrix and enhance tumor invasiveness, highlights the complex functions of desmogleins beyond autoimmunity.

## 1. Introduction

Oral Lichen Planus (OLP) is a chronic autoimmune mucocutaneous disorder, with a prevalence of 1% and 2% in the adult population [1,2,3,4]. From an epidemiological perspective, it affects women more frequently than men, particularly in the fourth and fifth decades of life [5,6,7].

The etiopathogenesis of OLP is multifactorial, likely triggered by the interaction between individual predisposition and external factors [5,7]. Accordingly, it is hypothesized that antigenic alterations in epithelial cells may be triggered by a range of biological, chemical, or pharmacological agents [7]. Stress and depression are considered a precipitating factor among the triggers, especially during episodes of disease exacerbation [8,9]. Additional exacerbating factors may involve particular classes of drugs and specific dental materials [5]. Recent studies suggest that genetics may contribute to the development of this disease. Specifically, variations in immune-related HLA antigens and genetic polymorphisms in the vitamin D receptor (VDR) seem to play a role in the pathogenesis of OLP [7]. Emerging evidence on the connection between oral microbiota and autoimmune diseases suggests that oral and/or gut dysbiosis may be directly involved in the development of OLP [10]. In addition, viral agents, such as HSV, HPV, and HCV, along with opportunistic pathogens like *Candida albicans*, are considered potential external triggers in the development of the disease [11,12,13,14]. Finally, in postmenopausal women, hormonal changes with increased circulating estrogen levels have been found to be responsible for a greater severity of oral lesions [5]. The pathogenesis of OLP is still widely debated, although it is believed that cellular immunity, mediated by CD4+ and CD8+ T lymphocytes, plays a fundamental role in the disease. Immune dysregulation may initiate a cascade of events, resulting in the activation of autoreactive T lymphocytes directed against keratinocytes [15,16]. Furthermore, the persistent course of the disease has been associated with Th17 and Tc17 cells, as well as IL-17A produced by T lymphocytes [1]. Clinically, OLP can be classified into quiescent and active forms. The quiescent forms include reticular, papular, and plaque-type lichen, whereas the active forms comprise atrophic/ulcerative and bullous variants [17]. These active forms are typically associated with pronounced symptoms and carry an estimated 5% increased risk of malignant transformation [1,5,7]. Symptoms may include pain, a burning sensation, dry mouth (xerostomia), altered taste (dysgeusia), and difficulty speaking [5]. The most frequently affected sites in OLP are the buccal mucosa, tongue, gums, vermilion border, and the mucosa of the lips [6,18,19]. The diagnostic process for OLP can be challenging due to its clinical and histopathological overlap with other conditions [5]. It encompasses both clinical and histological features, as summarized in Table 1 [20,21].

From a clinical perspective, erosive OLP can resemble Pemphigus Vulgaris (PV), as both autoimmune mucocutaneous diseases present with ulcerative-erosive lesions of the oral mucosa. However, in PV, bullous lesions and esophageal involvement are also observed, which are not seen in OLP [1]. OLP has traditionally been considered a cell-mediated condition, with antibodies not thought to play a role in its etiopathogenesis. However, recent scientific evidence indicates that in the atrophic and erosive forms of OLP, an autoimmune antibody response may target desmosomes and hemidesmosomes [25]. Some cases have shown that the typical antibodies of PV, anti-Dsg1, and anti-Dsg3, are present in patients with erosive OLP, which further complicates the differentiation between the two conditions. However, the role and significance of these antibodies in the erosive forms of OLP are still not fully understood [1]. It is hypothesized, however, that anti-Dsg antibodies may play a role in the onset of erosive OLP [25]. The aim of this scoping review is to examine the latest scientific evidence in the literature regarding the presence of anti-Dsg1 and anti-Dsg3 antibodies in patients with OLP, with a particular emphasis on their potential etiopathogenetic implications, as well as the similarities and differences with PV.

## 2. Materials and Methods

### 2.1. Study Registration

This study was registered on the Open Science Framework (OSF) on 13 April 2025 (https://osf.io/r6az5, accessed on 16 April 2025). This scoping review was conducted in accordance with the Preferred Reporting Items for Systematic Reviews and Meta-Analyses (PRISMA) extension for scoping reviews (Tables S1 and S2).

### 2.2. Objective

The aim of this scoping review is to examine the most recent scientific evidence on the presence of anti-Dsg1 and anti-Dsg3 antibodies in OLP and to explore the potential pathogenetic mechanisms underlying this overlap with PV.

### 2.3. Elegibility Criteria

For this scoping review, we included case reports, clinical conferences, clinical studies, clinical trials, controlled clinical trials, letters, multicenter studies, observational studies, randomized controlled trials, and human-based studies while excluding book chapters, systematic reviews, reviews, in vitro studies, and animal models. Furthermore, only studies published in English were considered. The review included all patients diagnosed with OLP who tested positive for anti-Dsg1 and anti-Dsg3 antibodies.

### 2.4. Search Strategy

The research was performed using MEDLINE/Pubmed, Ovid, and Scopus, applying search filters, such as (“Circulating antibodies” OR “autoantibodies”) AND (“Desmoglein 1” OR “DSG1”) AND (“Desmoglein 3” OR “DSG3”) AND (“Oral Lichen Planus” OR “OLP”), ((“Circulating antibodies” OR “serum antibodies” OR “autoantibodies”) AND (“Desmoglein 1” OR “DSG1”) AND (“Desmoglein 3” OR “DSG3”) AND (“Oral Lichen Planus” OR “OLP” OR “lichen planus of the oral mucosa”)), ((“Circulating antibodies” OR “serum antibodies” OR “autoantibodies”) AND (“Desmoglein 1” OR “DSG1”) AND (“Desmoglein 3” OR “DSG3”) AND (“Oral Lichen Planus” OR “OLP”) AND (“diagnosis” OR “pathogenesis” OR “biomarkers” OR “immunofluorescence” OR “ELISA”)). Articles published between 1990 and April 2025 were included in the analysis.

### 2.5. Study Selection and Data Extraction

A total of 32 articles were identified, of which 16 were duplicates, 2 were excluded because they were reviews, and 3 did not contain diagnoses of OLP with anti-Dsg1 and anti-Dsg3 antibodies. The data from the remaining 11 articles were included in a table (Table 2) based on variables such as diagnosis, age/sex, cutaneous clinical manifestations or involvement of other mucous membranes, localization of oral lesions, direct immunofluorescence (DIF) or indirect immunofluorescence (IIF), and Enzyme-Linked Immunoassay (ELISA) testing.

### 2.6. Risk of Bias Assessment

The risk of bias in the included studies was evaluated using the CASP (Critical Appraisal Skills Programme) tools. CASP was selected for its capacity to systematically assess methodological quality and potential bias across different study designs, ensuring a standardized evaluation of methodological rigor (Table S3).

## 3. Results

This scoping review was conducted by analyzing the literature from the MEDLINE/PubMed, Ovid, and Scopus databases using the following keywords with Boolean operators: (“Circulating antibodies” OR “autoantibodies”) AND (“Desmoglein 1” OR “DSG1”) AND (“Desmoglein 3” OR “DSG3”) AND (“Oral Lichen Planus” OR “OLP”); ((“Circulating antibodies” OR “serum antibodies” OR “autoantibodies”) AND (“Desmoglein 1” OR “DSG1”) AND (“Desmoglein 3” OR “DSG3”) AND (“Oral Lichen Planus” OR “OLP” OR “lichen planus of the oral mucosa”)); ((“Circulating antibodies” OR “serum antibodies” OR “autoantibodies”) AND (“Desmoglein 1” OR “DSG1”) AND (“Desmoglein 3” OR “DSG3”) AND (“Oral Lichen Planus” OR “OLP”) AND (“diagnosis” OR “pathogenesis” OR “biomarkers” OR “immunofluorescence” OR “ELISA”)).

Based on the analysis of the 11 selected studies, a database was created in which patients were categorized according to diagnosis, age/sex, cutaneous manifestations or involvement of other mucous membranes, involved oral mucosal sites, direct immunofluorescence (DIF) or indirect immunofluorescence (IIF) outcome, and Enzyme-Linked Immunoassay (ELISA) testing.

The analysis of these characteristics reveals that 27 (25%) patients with erosive OLP showed the positivity of anti-Dsg1 and Dsg3 antibodies.

Moreover, even in cases where the threshold value was not reached, anti-Dsg1 and Dsg3 antibodies were still present. In cases of reticular OLP, these antibodies were detected in all 54 cases, although their levels did not exceed the threshold required to be considered positive. Moreover, among the 103 patients whose sex was reported, 78 (80%) were women between their fourth and sixth decades of life. In 68% of cases, there were no reported cutaneous or other mucosal manifestations, making the oral cavity the exclusive site of involvement. Since the erosive form of OLP was the most represented (62.7%), the predominant clinical features were symptomatic erosions and ulcerations of the oral mucosa, with the buccal mucosa, tongue, and gingiva being the most commonly affected sites. However, cases of reticular OLP have also been documented (37.2%).

Data regarding DIF and IIF were inconsistent: in 11 cases, both tests were negative, while in 84 cases, no information about these examinations was provided at all.

However, in the studies by Raha et al. and Lukac et al., almost all patients who underwent DIF tested positive [26,27]. Finally, the most interesting finding is represented by the ELISA test, which showed positivity for anti-Dsg1 and/or anti-Dsg3 antibodies in the majority of the patients (93.2%). Even in samples that did not reach the threshold value for ELISA test positivity, the presence of these antibodies was still detected.

## 4. Discussion

OLP is an inflammatory disease that can affect both the skin and mucous membranes, which encompasses a wide range of oral clinical manifestations, from asymptomatic white lesions to painful erosive–ulcerative lesions [36,37,38]. This scoping review highlights that the erosive forms of OLP are more frequently associated with the presence of anti-Dsg1 and anti-Dsg3, leading to a clinical and immunoserological overlap with Pemphigus Vulgaris. Several studies had already hypothesized that the production of anti-Dsg1 and Dsg3 antibodies in OLP could develop as a result of humoral epitope spreading (ES) [1,39]. Epitope spreading (ES) refers to an autoimmune response initially directed against a single antigen, which subsequently expands to target additional antigens. In this context, ES is thought to occur through an intermolecular mechanism, resulting in the diversification of the autoimmune response toward multiple autoantigens located in anatomically adjacent tissues [1,39]. This could explain the immune response against desmogleins, but not the association with erosive OLP. It has been observed that, particularly in the hyperkeratotic forms of OLP, the persistent basal cell damage triggers the release of p53, which, in turn, induces apoptosis in keratinocytes. Over time, the sustained activity of p53, combined with the epithelium’s inability to maintain proper turnover, leads to the progression toward an atrophic form of OLP [40]. If the majority of basal keratinocytes were to undergo apoptosis, the regenerative compartment of the epithelium would be lost, inevitably leading to atrophy or ulceration. Excessive apoptosis, coupled with inefficient clearance of dying cells by phagocytes, may result in secondary necrosis and the release of autoantigens—including desmogleins—which can activate T and B cells, thereby triggering autoimmunity [11,25]. Didona et al. suggested that anti-Dsg antibody production occurs late in OLP progression and is associated with more aggressive forms. However, some authors argue these autoantibodies might not contribute to the disease’s pathogenesis since antibody levels were often positive but below the threshold [29,33,41,42]. Furthermore, as indicated by other studies, there seemed to be no direct association between anti-Dsg antibody levels and disease severity [25]. Another etiopathogenetic hypothesis was that it could be linked to a collateral and still poorly understood function of desmogleins [40,43]. In addition to their well-known adhesive properties, desmogleins accelerate the differentiation of keratinocytes above the basal layer [40,43,44].

Specifically, Dsg-1 suppresses the EGFR/Erb1 signaling pathway by inhibiting the formation of the Ras/Raf complex, thereby accelerating keratinocyte differentiation [40]. In patients with atrophic OLP, Desmoulins may have been released to promote keratinocyte differentiation and the upregulation of p53, and this antigenic expression could have led to the development of antibodies against desmogleins and autoimmunity. The link between desmogleins, particularly Dsg-3, and p53 was established in the study by Rehman et al. [45]. This research demonstrated how Dsg-3 acts as an “anti-stress” protein, capable of counteracting p53 in maintaining epithelial homeostasis [45]. Lukac et al., in their study, suggested another hypothesis: the increased antibody titers of anti-Dsg1 and Dsg3 found in patients with the erosive form of OLP may have triggered these atrophic-erosive forms [27]. This explained the clinical overlap with PV. However, Didona et al. dismissed this hypothesis after comparing the histological analysis of patients with erosive OLP who were positive for Dsg 1 and 3 with those who had PV [30]. The histological comparison showed that erosive OLP still exhibited the typical pathological features of Lichen, such as band-like infiltration, thickening of the granular layer, and apoptosis of basal keratinocytes (as reported in Table 3) [30]. In addition, the antibodies from patients with OLP did not induce acantholysis in vitro [30]. Finally, the authors concluded that from the Dispase-based Keratinocyte Assay (DDA), it was evident that the anti-Dsg antibodies in cases of OLP were not pathogenic and that their presence was a consequence of chronic antigen exposure induced by apoptotic basal cells [30].

Due to their pro-proliferative effect on keratinocytes, the presence of Dsg-1 and Dsg-3 in erosive forms of OLP has been hypothesized as a potential future marker of malignant transformation. These alterations are thought to reflect a characteristic feature associated with the loss of the basal cell phenotype [50]. Meliante et al. provided evidence suggesting a possible association between the presence of Dsg-3 and lymph node metastases in the neck from oral squamous cell carcinoma (OSCC), thereby supporting the aforementioned hypothesis [50,51].

A study conducted by Wan et al. showed that Dsg3 exhibited increased expression in OSCC and in SCC of other organsFine modulo [52,53]. Dsg3 may support the tumor microenvironment by influencing collagen deposition and facilitating tumor progression. Specifically, Dsg3 promoted the expression of MMP-13 (Matrix metalloproteinase 13), an enzyme that degraded ECM components, including the major types of collagen essential for tissue integrity, wound healing, and tissue homeostasis. In tumors, therefore, the increased activity of MMPs enhanced local invasiveness [52,53]. Therefore, a potential future approach could involve using the presence of these proteins as markers for the malignant progression of potentially evolving lesions, such as atrophic–erosive OLP [50]. However, a limitation for further studies on OLP was that DIF, IIF, and ELISA were performed in only a small percentage of patients, meaning that robust data on the presence of Dsg in OLP is still lacking.

Future research should focus on detecting these antibodies in larger population samples, possibly with a more detailed investigation of antibody titers across different forms of OLP. Therefore, large-scale studies are needed to advance and clarify the association between the presence of anti-Dsg antibodies and OLP. Finally, significant developments could emerge from a serological study on patients with Bullous Lichen, a form that clinically closely resembled PV.

## Figures and Tables

**Table 1:** Diagnostic criteria from OLP.

Clinical Criteria	Histopathological Criteria	Diagnostic Tools	
Diagnostic criteria of Oral Lichen Planus (OLP) and oral lichenoid lesions (OLL) as proposed by Van der Meij & Van der Waal, 2003 [22]			
Bilateral and symmetrical lesions. Reticular pattern of a lace-like network of slightly raised gray-white streaks. Erosive, atrophic, bullous, and plaque-like forms are diagnostic only when reticular lesions are also present elsewhere in the oral cavity. Lesions resembling OLP but do not fully meet the criteria, should be described as “clinically compatible with” OLP.	Dense band of immune cells, primarily lymphocytes, located in the uppermost layer of the connective tissue. Degeneration (liquefaction) of the basal layer of epithelial cells. No evidence of epithelial dysplasia. When the microscopic (histological) characteristics are not clearly defined, the phrase “histopathologically compatible with” should be used.	**Hypercheratosic** **OLP**	**Biopsy**
Diagnostic criteria for Oral Lichen Planus OLP according to the American Academy of Oral and Maxillofacial Pathology (AAOMP), 2016 [23]		Azzi L. et al., Chronic ulcerative stomatitis: A comprehensive review and proposal for diagnostic criteria. Oral Dis. 2019 [24]	
**Lesions are distributed in multiple areas symmetrically** Oral lesions can appear as white or red areas in different forms: reticular, atrophic, erosive, plaque-like, or bullous. **Lesions are not localized**. Not restricted to areas where smokeless tobacco is placed. Not specifically located near or in direct contact with dental restorations. **The appearance of the lesion is not associated with it**. The start of a medication. The use of products containing cinnamon.	A dense lymphocytic band, continuous or patchy, is present at the epithelial–connective tissue interface. Degenerative changes (liquefaction/hydropic degeneration) in the basal cell layer. Presence of lymphocytes migrating into the epithelial layer (lymphocytic exocytosis). There are no signs of epithelial dysplasia. There is no evidence of verrucous-type changes in the epithelial structure.	**Atrophic/Erosive** **OLP**	**Biopsy** **DIF, IIF, ELISA** (Differential Diagnosis of Chronic ulcerative stomatitis)

**Table 2:** Characteristics of the patients included in the review.

Study ID	Patients with OLP	Age/Sex	Skin/Mucosal Findings	Involved Oral Sites	DIF/IIF	ELISA-Anti-Dsg1 (RU/ML) Negative < 20.0 Positive > 20.0
Raha S et al. Oral Surg Oral Med Oral Pathol Oral Radiol. 2023 Sep; 136(3): 353–359 [26].	1	61/M	Absent	Erosive lesions seen on the soft palate, both buccal mucosa	DIF: positive	Anti-Dsg1 16.1 (RU/ML) Anti-Dsg3 19 (RU/ML)
	1	30 M	Absent	Oral ulcerations seen on the left buccal mucosa and the left lateral border of the tongue.	DIF: positive	Anti-Dsg1 14 (RU/ML) Anti-Dsg3 18 (RU/ML)
	1	45/F	Absent	Oral ulcerations seen on the left buccal mucosa and soft palate	DIF: positive	Anti-Dsg1 7.6 (RU/ML) Anti-Dsg3 8 (RU/ML)
	1	60/M	Absent	Ulcerative lesion seen on attached gingiva in 45 region and right buccal mucosa	DIF: positive	Anti-Dsg1 9 (RU/ML) Anti-Dsg3 14.9 (RU/ML)
Lukac J. et al., Croat Med J. 2006 Feb; 47(1): 53–8 [27].	32 Erosive LP	30–72 yrs M/F 7/25			IIF: 18/22 Positive/tested	Anti-Dsg1 10.4 (4.9–16.3) (RU/ML) Anti-Dsg3 9.1 (3.3–12.9) (RU/ML)
	25 Reticular LP	20–52 yrs M/F 5/20			IIF: 3/15 Positive/tested	Anti-Dsg1 2.3 (1.5–4.9) (RU/ML) Anti-Dsg3 2.4 (1.1–3.1) (RU/ML)
Giurdanella F. et al., Front Immunol. 2018 Apr 24; 9: 839 [28].	10					
Herrero-González JE et al., Int J Dermatol. 2016 Jun; 55(6): 634–9 [29].	21 15-Erosive LP	56 yrs M/F 5/16	Other mucosal: 7 Genital Skin: 4	Oral ulcerations	IIF: 1/positive-tested	Anti-Dsg3: 1 positive
Didona D. et al., J Dtsch Dermatol Ges. 2024 Oct; 22(10): 1392–1399 [30].	4-Erosive LP			Oral erosion	DIF-IIF: 4/negative-tested	Anti-Dsg3: 4 positive Anti-Dsc2-3: 3 positive
Saad I. et al., J Contemp Dent Pract. 2018 Oct 1; 19(10): 1204–1213 [31].	20 Erosive LP	36–57 yrs M/F 6/14		Buccal mucosa (100%), tongue (50%), lip (25%), alveolar mucosa (15%), retromolar area (5%)		Anti-Dsg1: 16 positive 8.40–130.30 (48.88 ± 37.87) (RU/ML) Anti-Dsg3: 19 positive 6.65–43.55 (21.59 ± 11.81) (RU/ML)
Vahide L. et al., Arch Dermatol Res. 2017 Sep; 309(7): 579–583 [25].	53 Erosive OLP:24 Reticular OLP:29					Anti-Dsg1 Erosive OLP: 1.9 (1.7, 2.3) (RU/ML) Reticular OLP: 1.9 (1.6, 2) (RU/ML) Anti-Dsg3 Erosive OLP: 3 (2, 5.4) (RU/ML) Reticular OLP: 2 (2, 3.6) (RU/ML)
Kinjyo C. et al., J Dermatol. 2015 Jan; 42(1): 40–1 [32].	1: OLP	43 yrs M/1		Oral erosion	DIF: negative IIF: negative	Anti-Dsg1: 49 (RU/ML) Anti-Dsg3 36 (RU/ML)
Muramatsu K. et al., J Dermatol. 2016 Nov; 43(11): 1350–1353 [33].	2: Erosive OLP	68–85 yrs F/2		Erosion on Buccal mucosa, gingiva, tongue	DIF: negative IIF: negative	Anti-Dsg1: 47–12 (RU/ML) Anti-Dsg3: 34–19 (RU/ML)
Rambhia KD. Et al., India. Indian J Dermatol Venereol Leprol. 2018 Nov-Dec; 84(6): 667–671 [34].						Anti-Dsg1: 19 (RU/ML) Anti-Dsg3: 16 (RU/ML)
Gholizadeh N. et al., Iran J Pathol. 2015 Spring; 10(2): 136–40 [35].	35	43.7 (23–60 yrs)	Absent		IID: positive-tested	Anti-Dsg1: 6.9 (0–15.6) (RU/ML) Anti-Dsg3: 30.32 (RU/ML)

**Table 3:** Differences between atrophic–erosive Oral Lichen Planus and Pemphigus Vulgaris.

	Atrophic/Erosive Oral Lichen Planus	Pemphigus Vulgaris
**Etiology**	T-cell-mediated immunity (CD8+, Langerhans cells, macrophages)	IgG autoantibodies against desmogleins
**Target**	Basal keratinocytes (apoptosis induced by CD8+ T cells)	Desmoglein 3 (and sometimes Dsg1) in desmosomes of keratinocytes
**Localization**	Vestibular mucosa, tongue, and gingiva (bilateral and symmetric)	All areas of the oral mucosa
**Pathological features**	Band-like lymphocytic infiltrate, liquefaction degeneration of basal layer	Suprabasal acantholysis, “tombstone” appearance, no band-like infiltrate
**Oral Clinical Features**	Erythematous and ulcerated areas bordered by reticular striae	Extensive oral lesions that significantly impair food intake.
**Skin/Other Mucosal**	Skin lesions are dark red, papular, and roundish in shape. Esophageal involvement is usually asymptomatic. In women, vulvo-vagino-gingival syndrome may occur, while in men, peno-gingival syndrome can manifest.	Patients may develop flaccid blisters on the skin and cutaneous erosions. Odynophagia occurs in cases of esophageal erosions.
**Direct Immunofluorescence (DIF)**	Fibrinogen deposits with a fringed appearance in the basement membrane zone, IgM.	Reticular deposition of immunoglobulins (IgG or IgM) or C3 in the intercellular spaces.
**Indirect Immunofluorescence (IIF)**	Almost always negative, although positive cases have been documented.	Positive: anti-Dsg3 and/or anti-Dsg1 antibodies.
**ELISA**	It can be positive for the presence of anti-Dsg3 and/or anti-Dsg1 antibodies; however, the antibody titer is often low and does not reach the threshold value.	Positive: anti-Dsg3 and/or anti-Dsg1 antibodies [1,7,30,39,46,47,48,49].

## Data Availability

The original contributions presented in this study are included in the article/Supplementary Materials. Further inquiries can be directed to the corresponding author.

## Supplementary Materials

The following supporting information can be downloaded at: https://www.mdpi.com/article/10.3390/antib14020051/s1, Table S1: PRISMA statement; Table S2: Flow chart; Table S3: Risk of bias. Refs. [54,55,56] are cited in Supplementary Materials.
