# Supplementary material for: Circulating Antibodies Against DSG1 and DSG3 in Patients with Oral Lichen Planus: A Scoping Review
Source: Antibodies (Basel). 2025 Jun 18;14(2):51. doi: 10.3390/antib14020051 (PMC12189673; doi:10.3390/antib14020051)
Supplement: Supplementary file 1 [file antibodies-14-00051-s001.zip › antibodies-3623898-supplementary/S2.pdf]

PRISMA Flow Diagram for the scoping review process

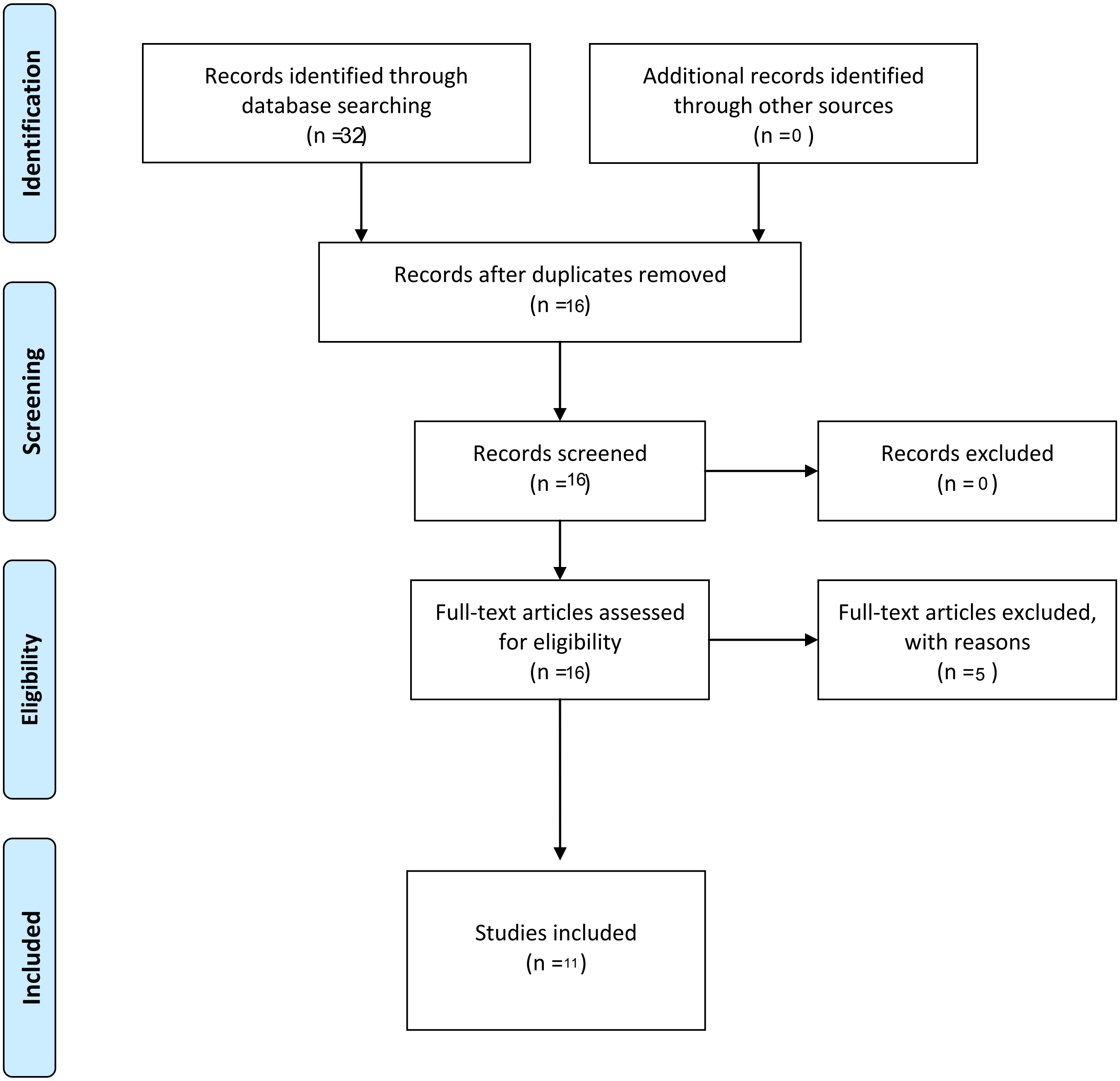

From: Moher D, Liberati A, Tetzlaff J, Altman DG, The PRISMA Group (2009). *P*referred *R*eporting *I*tems for *S*ystematic Reviews and *M*eta-*A*nalyses: The PRISMA Statement. PLoS Med 6(7): e1000097. doi:10.1371/journal.pmed1000097
Modified: The Joanna Briggs Institute Reviewers' Manual 2015. Methodology for JBI Scoping Reviews. Published by the Joanna Briggs Institute, 2015.
For more information, visit: www.prisma-statement.org.
http://joannabriggs.org/assets/docs/sumari/Reviewers-Manual_Methodology-for-JBI-Scoping-Reviews_2015_v2.pdf
