# Supplementary material for: Circulating Antibodies Against DSG1 and DSG3 in Patients with Oral Lichen Planus: A Scoping Review
Source: Antibodies (Basel). 2025 Jun 18;14(2):51. doi: 10.3390/antib14020051 (PMC12189673; doi:10.3390/antib14020051)
Supplement: Supplementary file 1 [file antibodies-14-00051-s001.zip › antibodies-3623898-supplementary/S3.pdf]

CASP Checklist:
For systematic reviews with meta-analysis of observational studies

Reviewer Name:	
Paper Title:	Circulating Antibodies Against DSG1, and DSG3 in patients with Oral Lichen Planus: A Scoping Review
Author:	Domenico De Falco 1,*, Francesca Iaquinta 1, Doriana Pedone 1, Alberta Lucchese 2, Dario Di Stasio 2 and Massimo Petruzzi 1
Web Link:	
Appraisal Date:	

During critical appraisal, never make assumptions about what the researchers have done. If it is not possible to tell, use the "Can't tell" response box. If you can't tell, at best it means the researchers have not been explicit or transparent, but at worst it could mean the researchers have not undertaken a particular task or process. Once you've finished the critical appraisal, if there are a large number of "Can't tell" responses, consider whether the findings of the study are trustworthy and interpret the results with caution.

Section A: Is the basic study design valid for a systematic review?

1. Did the systematic review address a clearly formulated research question?	[x] Yes [ ] No [ ] Can't Tell

CONSIDER:
Did the researchers state a research question and a null hypothesis?
For a systematic review of observational studies, a research question can be 'formulated' in terms of the PECOT(S) framework:

- *Population*
- *Exposure/Risk factor*
- *Detection of a beneficial or harmful effect*
- *Comparator/Controls*
- *Outcome/s or Event/s*
- *Time, e.g., length of time in which to detect outcomes or events, or time of exposure*
- *Setting*

2. Did the researchers search for appropriate study design(s) to answer the research question?	[X] Yes [ ] No [ ] Can't Tell

CONSIDER:
If the research question is concerned with the identification of risk factors or exposures associated with a particular event or outcome, observational studies are appropriate study designs to address the research question in a systematic review, for example:

- *Cohort studies follow a group of people who share a common characteristic or exposure over time and compare them with another group who do not have that characteristic or exposure.*
- *Case-control studies compare a group of people who have a specific outcome or condition (cases) with a group of people who do not have it (controls) and look for differences in their past exposures or risk factors.*
- *Cross-sectional studies measure the prevalence of a characteristic, outcome, or exposure in a population at a single point in time or over a short period.*

Notes to support interpretation of Section A, Questions 1 and 2:
If you answered "No" to both these questions:

- It is likely that the researchers did not clearly formulate the fundamental aspects of the research question, and the most appropriate way of answering it. If this is the case, it is likely other problems will arise during the conduct of the systematic review
- Consider whether it would be useful to continue with the critical appraisal process

Section B: Is the systematic review methodologically sound?

3. Were all the relevant primary research studies likely to have been included in the systematic review?<br><br>a) Searching for primary research studies	[x] Yes [ ] No [ ] Can't Tell

CONSIDER:

- *Was the search strategy comprehensive and clearly reported?*
- *Did the search include 1 or more of the major bibliographic databases, e.g., MEDLINE/PubMed, and Embase?*
- *Did the researchers provide MESH terms for MEDLINE, or their equivalent for other databases?*
- *Were relevant subject-specific bibliographic databases searched?*
- *Did the search include non-English language studies?*
- *Did the researchers undertake citation searching, including hand-searching of reference lists from primary research studies included in the systematic review?*
- *Did the search include unpublished studies? For instance, did the search include registers of ongoing trials or preprint repositories?*
- *Did the researchers consult experts in the field about potential primary research studies or ongoing trials that could be included?*

b) Screening primary research studies from the search	[x] Yes [ ] No [ ] Can't Tell

CONSIDER:

- *Did the researchers define appropriate eligibility or inclusion and exclusion criteria for the research question?*
- *Did the researchers design and implement a robust process to screen the primary research studies? For instance, two researchers working independently, with a third independent researcher to resolve any disagreements.*
- *Was screening based on title and abstract of primary research studies found during the search?*
- *Did the researchers adhere to the eligibility criteria?*

c) Selecting primary research studies to include in the systematic review	[x] Yes [ ] No [ ] Can't Tell

CONSIDER:

- *Did the researchers design and implement a robust process to select the primary research studies according to the eligibility criteria? For instance, two researchers working independently, with a third independent researcher to resolve any disagreements.*

• *Were decisions to include or exclude primary research studies based on full-text analysis?*<br>• *Did the researchers adhere to the eligibility criteria?*<br>• *Was the level of agreement between the researchers responsible for selecting the primary research studies calculated and reported? For instance, by calculating the kappa statistic of inter-rater reliability?*	
d) Summarising the search and its outputs	☑ Yes ☐ No ☐ Can't Tell
CONSIDER:<br>*Did the researchers present a PRISMA-type flowchart, including the numbers of primary research studies that were:*<br>• *Duplicates?*<br>• *Screened out?*<br>• *Excluded, with the reasons for exclusion?*<br>• *Included in the systematic review?*<br>• *Included in the meta-analysis (data may not have been complete in some of the primary research studies)?*	
4. Did the researchers assess the validity or methodological rigour of the primary research studies included in the systematic review?	☑ Yes ☐ No ☐ Can't Tell
CONSIDER:<br>*Lack of methodological rigour in the individual primary research studies can affect the validity and interpretation of the findings of the systematic review with meta-analysis.*<br>• *Did the researchers use a validated tool to assess the methodological rigour of the primary research studies included in the systematic review?*<br>• *Was the tool appropriate to assess the type(s) of study design(s) included in the systematic review? For example, for case-control and cohort studies, the Newcastle-Ottawa Scale or the ROBINS-E tool.*<br>• *Did the researchers present the findings from their quality assessment in sufficient detail, and interpret them accurately?*	
5. Did the researchers extract, and present information from the individual primary research studies appropriately and transparently?<br><br>(a) Extraction of data	☑ Yes ☐ No ☐ Can't Tell

CONSIDER:<br>• *Did the researchers design and implement a robust process for the extraction of data from the individual primary research studies?*<br>• *Did the researchers follow guidance on data extraction?*<br>• *Did the researchers use a standardised form or software programme to record the data to ensure completeness and accuracy?*<br>• *Did the researchers extract the relevant data for the study-level characteristics and the results of each primary research study?*	
(b) Presentation of data	☒ Yes ☐ No ☐ Can't Tell
CONSIDER:<br>• *Did the researchers present the key characteristics of the individual primary research studies, e.g., in a table? For instance, the number of participants, the profile of participants (age, sex), the intervention, the comparator, the outcome/s evaluated, and the study timeframe.*<br>• *Did the researchers present the results of the individual primary research studies in a Forest plot or combination of table and Forest plot? For instance, the effect size/s, the confidence-interval ranges, and the P values. NB: The Forest plot should also show the overall result from the meta-analysis*	
Notes to support the interpretation of Section B, Questions 3-5:<br>If you answered "No" to these questions, it is likely that there is a lack of methodological rigour in the conduct of the systematic review, which means it is best to interpret the results with caution, and to assess how those aspects of poor methodology will have an impact on the results of the systematic review.<br>• For *Question 3,* a "No" response indicates that this systematic review may have missed primary research studies that could have contributed to answering the research question; in a systematic review with meta-analysis, the results of any missing primary research studies could have altered the effect estimate for the systematic review.<br>• For *Question 4*, a "No" response indicates that the researchers did not identify any systematic bias or confounding factors in the primary research studies that could have affected the results of the systematic review; in the absence of this information, it is not possible for you to assess in what ways the results of the systematic review could have been affected, and it is best to be cautious when interpreting the results.<br>• For *Question 5*, a "No" response indicates that the researchers did not organise the data from the primary research studies in a coherent way such that it could analysed appropriately, and thereby reliable conclusions drawn from it.<br>If you answered "No" to all three questions in Section B, consider whether it would be useful to continue with the critical appraisal process.	
Section C: Are the results of the systematic review trustworthy?	
6. Did the researchers analyse the pooled results of the individual primary research studies appropriately?	☒ Yes ☐ No ☐ Can't Tell

CONSIDER:<br>• *Did the researchers undertake a sample-size estimation during the design and planning of the systematic review?*<br>• *Did the number of participants whose outcomes were entered into the analysis meet that estimation, i.e., was the sample size sufficient to detect any effect on the outcomes of interest?*<br>• *Did the researchers use an appropriate effect measure?*<br>• *Did the researchers provide confidence-interval ranges for the effect estimates in the systematic review?*<br>• *Did the researchers provide p values for the effect estimates in the systematic review?*<br>• *Did the researchers provide a minimal important difference, that is the smallest possible difference in outcome that would be meaningful to people experiencing the exposure or risk factor?*<br>• *Did the researchers assess the level of statistical heterogeneity (variability) among the primary research studies? For example, using the I^2 statistic.*<br>• *Did the researchers use an appropriate model of meta-analysis for the level of heterogeneity among the primary research studies (a random-effects model if there was heterogeneity or a fixed-effects model if the primary research studies were all investigating the same underlying effect)?*<br>• *Did the researchers perform any sensitivity analyses?*<br>• *Did the researchers analyse the reasons for heterogeneity using subgroup analysis or meta-regression? For subgroup analysis, see Question 6.1, and for meta-regression see Question 6.2.*<br>• *Did the researchers investigate the small-study-effect, and assess the potential for publication bias in the systematic review (e.g., using a funnel plot)?*	
6.1 Subgroup analysis	Yes [X] No [ ] Can't Tell
CONSIDER:<br>*Were the characteristics or effect modifiers for investigation:*<br>• *Specified in the study protocol, with the direction of effect, and statistical tests to be used?*<br>• *Clearly defined, with a rationale for selection?*<br>• *Not closely related to other characteristics, i.e., differentiation is possible?*<br>• *Analysed in relation to the primary outcome?*<br>*If continuous data were allocated to categories, were the thresholds or cut-off points specified in the study protocol together with a rationale?*<br>*If a large number of characteristics were investigated, or subgroup analyses conducted, did the researchers adjust for multiple testing?*<br>*Was a test for interaction undertaken to determine whether any subgroup effects were statistically significant?*<br>*Was the analysis of effect modification based on comparison within rather than between studies?*	
6.2 Meta-regression	Yes [ ] No [X] Can't Tell

CONSIDER:
Were the characteristics or effect modifiers for investigation:
- *Specified in the study protocol, with the direction of effect?*
- *Continuous data? If continuous data were allocated to categories, were the thresholds or cut-off points specified in the study protocol with a rationale for selection?*

If a large number of characteristics or effect modifiers were investigated, or meta-regression analyses performed, did the researchers adjust for multiple testing?
Was a test for interaction undertaken to determine whether any effects were statistically significant?
Was a random-effects model used for the meta-regression analyses?
Was the analysis of effect modification based on comparison within rather than between studies?

7. Did the researchers report any limitations of the systematic review and, if so, do the limitations discussed cover all the issues you have identified during critical appraisal?	[X] Yes [ ] No [ ] Can't Tell

CONSIDER:
- *Did the researchers comment on the size of the sample in the meta-analysis and whether it was large enough to detect an effect of the exposure or risk factor if there was one?*
- *Did the researchers consider the appropriateness of the effect measure or measures they used?*
- *Did the researchers reflect on the precision of the results of the systematic review, i.e., the confidence-interval range? The smaller the range, the narrower the confidence intervals, meaning the result is more precise, and closer to the true effect size.*
- *If relevant, did the researchers note whether the confidence-interval range included the "line of no effect" (0 for a difference, 1 for a ratio, where the null hypothesis holds true), or whether the lower limit of the confidence-interval range was close to the "line of no effect", and discuss the implications for the results of the meta-analysis?*
- *If the results were statistically significant (i.e., they were less likely to be due to chance), did the researchers discuss whether the results would be important or meaningful for the outcomes experienced by individuals and/or populations using a minimal important difference specific to the research question?*
- *Did the researchers consider whether relevant primary research studies could have been missed?*
- *Did the researchers mention any systematic bias identified during the risk-of-bias/quality assessment of the primary research studies, and explain how it might have influenced the effect estimate in the meta-analysis?*
- *Did the researchers mention any potential sources of confounding that could have influenced the effect estimate in the meta-analysis?*
- *Did the researchers discuss the implications of any sensitivity analyses?*
- *Did the researchers discuss the impact of the level of heterogeneity on the results of the meta-analysis?*
- *Did the researchers investigate the reasons for any heterogeneity across the primary research studies and discuss the implications? For subgroup analysis, see Question 7.1, and for meta-regression, see Question 7.2.*
- *Did the researchers discuss the effect of any publication bias on the results of the meta-analysis?*

7.1 Subgroup analysis	[X] Yes [ ] No [ ] Can't Tell

CONSIDER:<br>• *If characteristics or effect modifiers were not pre-specified, did the researchers address whether bias was introduced into the analysis?*<br>• *Did the researchers reflect on whether the characteristics or effect modifiers selected were well-defined to ensure clarity about the effect being investigated?*<br>• *If no rationale was given for the selection of specific characteristics or effect modifiers, or the rationale was not supported by evidence or a plausible argument of meaningfulness, did the researchers discuss whether this affected the validity or relevance of the subgroup analysis?*<br>• *If characteristics or effect modifiers were closely related to other characteristics, did the researchers mention the potential for confounding?*<br>• *Did the researchers outline whether the sample sizes in any subgroup analyses were sufficient to detect an effect of the exposure or risk factor on the primary outcome?*<br>• *If continuous data were allocated to categories, did the researchers address whether the thresholds or cut-off points could have introduced bias into the subgroup analysis or were not meaningful either clinically or in terms of public and population health?*<br>• *If more than three characteristics or effect modifiers were investigated or subgroup analyses performed, did the researchers adjust for multiple testing and consider the potential to generate Type I errors?*<br>• *Did the researchers explain the results of any tests for interaction, and whether they were statistically significant?*<br>• *Did the researchers discuss the implications of whether the results of tests for interaction were quantitative or qualitative?*<br>• *If the analysis of effect modification was based on a comparison between studies, did the researchers reflect on whether the number of studies in the smallest subgroups was large enough for the results to be credible?*	
7.2 Meta-regression	☐ Yes ☒ No ☐ Can't Tell
CONSIDER:<br>• *If characteristics or effect modifiers were not pre-specified, did the researchers address whether bias was introduced into the analysis?*<br>• *If continuous data were allocated into categories, did the researchers address whether any thresholds or cut-off points for categorisation were arbitrary and could have introduced bias into the meta-regression or whether they were not meaningful clinically and/or in terms of public and population health?*<br>• *If more than three characteristics or effect modifiers were investigated, or meta-regression analyses performed, did the researchers adjust for multiple testing and consider the potential to generate Type I errors?*<br>• *Did the researchers discuss the implications of any tests for interaction and whether they were statistically significant?*	

• *If a random-effects model was not used to account for residual heterogeneity and/or mixed effects, which would have allowed for both within-study and between-study variation, did the researchers outline the implications for the results?*<br>• *If the analysis of effect modification was based on a between-study comparison, did the researchers reflect on whether the number of primary research studies in the meta-regression was sufficient for the results to be credible?*	
8. Would the benefits of acting upon the results outweigh any potential disadvantages, harms and/or additional demand for resources associated with acting on the results?	☒ Yes ☐ No ☐ Can't Tell
CONSIDER:<br>• *Are you clear about the likely benefits of acting upon the results bearing in mind the potential impacts of any study limitations?*<br>• *Did the researchers identify any potential disadvantages, unwanted outcomes, or negative impacts of acting on the results of the systematic review?*<br>• *If so, did the researchers assess any benefits against the disadvantages, unwanted outcomes, or negative impacts, and discuss the overall balance between benefit and harm?*<br>• *Did the researchers report any information on the potential demand for resources (e.g., cost, workforce, time, skills levels/skill mix, training needs, data collection and analysis, IT requirements) that might be associated with acting on the results of the systematic review?*	
• Notes to support interpretation of Section C, Questions 6, 7 & 8: If you answered "No" to these questions, it is likely that the researchers did not analyse and interpret the information from the primary research studies appropriately, nor did they discuss the limitations of the systematic review as fully as possible so it is not possible for you to assess the trustworthiness (validity and credibility) of the results of the systematic review. Finally, if there is no information on the likely resource demands of intervention, it is not possible for you to judge whether you have the resource capacity to act upon the results.<br>If you answered "No" to all three questions in Section C, consider whether it would be useful to continue with the critical appraisal process.	
Section D: Are the results of the systematic review relevant locally?	
9. Can the results of the systematic review be applied to your local population/in your local setting or context?	☒ Yes ☐ No ☐ Can't Tell
CONSIDER:<br>• *Are there differences between your local population and the participants in the primary research studies in the systematic review that would influence whether you would act upon the results?*<br>• *Are there differences between your local setting and the settings or contexts in the primary research studies in the systematic review that would influence whether you would act upon the results?*<br>• *Are there any outcomes or other factors that the researchers could have studied that would have been useful to you bearing in mind the needs of your local population and/or setting?*	
Notes to support interpretation of Section D, Question 9:	

• If you answered “No” to this question, it is not necessary to answer Question 10 because, irrespective of a systematic review’s methodological rigour, the results are not applicable to the individuals or populations for whom you are responsible.<br>• If you answered “Yes” to Question 9, answer Question 10	
Section E: Will the implementation of the results represent greater value for your service users or population?	
10. If actioned, would the findings from the systematic review represent greater or additional value for the individuals or populations for whom you are responsible?	☐ Yes ☐ No ☒ Can’t Tell
CONSIDER:<br>*Value equals the Outcome/s (Benefit minus Harm) divided by the Resources required for implementation.*<br>• *What resources would be needed to take action on the findings of the systematic review? Take account of various types of resource, not only costs, but also time, skills mix, skills development or training needs, IT requirements, and other material resources.*<br>• *If necessary, are you able to disinvest resources from other activities to be able to re-invest in actioning the findings from the systematic review?*	
Notes to support interpretation of Section E, Question 10:<br>• *If you answered “No” to this question, it is likely that the findings of the systematic review will not confer greater or additional benefit or value on the individuals and/or populations for whom you are responsible, despite the systematic review’s applicability to your local setting.*<br>• *If you answered “Yes” to the question, it is likely that the findings of the systematic review will confer greater or additional benefit or value on the individuals and/or populations for whom you are responsible, and you need to discuss with colleagues whether it would be appropriate to implement the findings in your local setting.*	

What is your conclusion about the systematic review – can it be used to support evidence-based decision-making?	☐ Yes ☐ No ☒ Can’t Tell
CONSIDER:<br>• *Would you use it to change practice or to recommend changes to care policy and procedures in your organisation?*<br>• *Could you judiciously take action on the information about the exposure or risk factor without delay?*	

CASP General SR Checklist: Collation of critical appraisal responses

Yes	Checklist question	Can't tell	No
	A. Is the basic study design valid for a systematic review?		
X	1. Did the systematic review address a clearly formulated research question?		
X	2. Did the researchers search for appropriate study designs to answer the research question?		
	B. Is the systematic review methodologically sound?		
X	3. Were all relevant primary research studies likely to have been included in the systematic review?		
X	4. Did the researchers assess the validity or methodological rigour of the primary research studies included in the systematic review?		
X	5. Did the researchers extract, and present information on the individual primary research studies appropriately and transparently?		
	C. Are the results of the systematic review trustworthy?		
X	6. Did the researchers analyse the results of the individual primary research studies appropriately?		
X	7. Did the researchers report any limitations of the systematic review and, if so, do the limitations discussed cover all the issues in your critical appraisal?		
X	8. Would the benefits of acting upon the results outweigh any potential disadvantages, harms and/or additional demand for resources associated with acting on the results?		
	D. Are the results of the systematic review relevant locally?		
X	9. Can the results of the systematic review be applied to your local population/in your local setting or context?		
	**E. Will the implementation of the results represent greater value for your service users or population?**		
	10. If actioned, would the findings from the systematic review represent greater or additional value for the individuals or populations for whom you are responsible?	X	

**APPRAISAL SUMMARY**: *List key points from your critical appraisal that need to be considered when assessing the validity of the results and their usefulness in decision-making.*		
**Positive/Methodologically sound**	**Negative/Relatively poor methodology**	**Unknowns**
homogeneity of results<br>methodological rigor	Small patient sample	

Referencing recommendation:

CASP recommends using the Harvard style referencing, which is an author/date method. Sources are cited within the body of your assignment by giving the name of the author(s) followed by the date of publication. All other details about the publication are given in the list of references or bibliography at the end.

Example:

Critical Appraisal Skills Programme (2024). CASP (insert name of checklist i.e. systematic reviews with meta-analysis of observational studies Checklist.) [online] Available at: insert URL. Accessed: insert date accessed.

Creative Commons

©CASP this work is licensed under the Creative Commons Attribution – Non-Commercial- Share A like. To view a copy of this licence, visit https://creativecommons.org/licenses/by-nc-sa/4.0/

Need further training on evidence-based decision making? Our online training courses are helpful for healthcare educational researchers and any other learners who:

- Need to critically appraise and stay abreast of the healthcare research literature as part of their clinical duties.
- Are considering carrying out research & developing their own research projects.
- Make decisions in their role, whether that be policy making or patient facing.

Benefits of CASP Training:

⇒ Affordable – courses start from as little as £6
⇒ Professional training – leading experts in critical appraisal training
⇒ Self-directed study – complete each course in your own time
⇒ 12 months access – revisit areas you aren't sure of and revise
⇒ CPD certification - after each completed module

Scan the QR code below or visit https://casp-uk.net/critical-appraisal-online-training-courses/ for more information and to start learning more.
